# Ten simple rules for making a software tool workflow-ready

**DOI:** 10.1371/journal.pcbi.1009823

**Published:** 2022-03-24

**Authors:** Paul Brack, Peter Crowther, Stian Soiland-Reyes, Stuart Owen, Douglas Lowe, Alan R. Williams, Quentin Groom, Mathias Dillen, Frederik Coppens, Björn Grüning, Ignacio Eguinoa, Philip Ewels, Carole Goble

**Affiliations:** 1 Department of Computer Science, The University of Manchester, Manchester, United Kingdom; 2 Melandra Limited, Stockport, United Kingdom; 3 Informatics Institute, University of Amsterdam, Amsterdam, The Netherlands; 4 Research IT, IT Services, University of Manchester, Manchester, United Kingdom; 5 Meise Botanic Garden, Meise, Belgium; 6 Department of Plant Biotechnology and Bioinformatics, Ghent University, Ghent, Belgium; 7 VIB Center for Plant Systems Biology, Ghent, Belgium; 8 Bioinformatics Group, Department of Computer Science, Albert-Ludwigs-University Freiburg, Freiburg, Germany; 9 Science for Life Laboratory (SciLifeLab), Department of Biochemistry and Biophysics, Stockholm University, Stockholm, Sweden; Dassault Systemes BIOVIA, UNITED STATES

## Introduction

In recent years, the volumes of data to be analyzed, as well as the complexity of that analysis, across many scientific fields (from genomics through to exoplanet exploration) have increased massively. As a consequence, *workflows* (abstract representations of data flow between multiple analytic tools) have become a core part of computational scientific analysis [1]. Automated computational workflows multiply the power of researchers, potentially turning “hand-cranked” data processing by informaticians into robust factories for complex research output [2].

However, in order for a piece of software to be usable as a workflow-ready tool, it may require alteration from its likely origin as a stand-alone tool. Research software is often created in response to the need to answer a research question with the minimum expenditure of time and money in resource-constrained projects [3,4]. The level of quality might range from “it works on my computer” to mature and robust projects with support across multiple operating systems [3].

Although our suggestions in this article are mostly implementable with minor changes to the software, authors should not be afraid to rewrite their code, even substantially. We recommend following established engineering practices for research software [5,6], such as establishing continuous testing to ensure that code refactoring does not cause unintended changes.

Despite significant increase in uptake of workflow tools [7], there is little specific guidance for writing software intended to slot in as a tool within a workflow, or on converting an existing stand-alone research-quality software tool into a reusable, composable, well-behaved citizen within a larger workflow.

We therefore here introduce 10 simple rules for developers of research software, which we argue will enable their computational tools to be fully utilized in modern workflow management systems, but also improve their maturity and use in other environments. As many of these rules represent good practice for software development in general, the guidance presented is likely to be of use to software developers across the wider community.

Note that while we may give examples below, we have tried to not give specific technical recommendations (i.e., language, platform, engine choice), as technology choices need to be informed by requirements specific to a research domain, computational infrastructures, and related tool ecosystems. For further guidance on workflow technologies in bioinformatics, we refer to [8,9].

## Rule 1: Make sure a workflow engine can talk to your software easily

The fundamental notion of a computational workflow is that a workflow engine runs software tools in a given sequence, feeding data from one step to the next [8]. As such, all input and output data should be computer-addressable—this could mean by a standard stream, or a file system, or the body of a http packet, or a FAIR digital object [10]. It’s usually simplest to use a command line application to achieve this.

Every option for how your software runs should be *configurable* at runtime, so each of these options should be exposed by the software’s interface. If your software relies on configuration files or hard-coded values, it should be possible to override each configuration option with runtime arguments, for example, through arguments to a command line application. Specific advice on per-user config files is expanded on in Rule 8.

Your input and output file(s) or directory(ies) should always be specified as *arguments* at runtime. It is much easier to build workflows that can pass data between steps by explicit file path, rather than workflows having to infer file paths by convention (for example, taking the newest file from a preconfigured results directory). Command line tools typically output status information to the console and should follow usual convention for standard streams [11–13] for ease of development.

Crucially, one should not expect software to automate well simply because it is mature and from a reliable source—a prime example of this is Microsoft Office, which Microsoft specifically advises is not supported to be run unattended in a robotic process automation (RPA) environment [14].

## Rule 2: Make your tool simple to install

As an author of a software tool, it’s likely that you want to share the tool with a diverse user base, so it’s important to value their time, and consider their potential frustrations. As a consumer of software, it’s common to spend hours or even days of frustration just installing new tools. Removing this initial challenge for new users is likely to boost uptake of your software; your user’s choice of software is likely to be led by its ease of use [15].

Instructions such as “create MySQL table X and copy Y to Z” are frustrating, slow, and error-prone for a user, and such processes are easy to automate. Ideally, someone with the skills typical of a researcher who wants to try your software tool should be able to install it automatically, by using a package manager to install from a registry, such as Yum [16], Conda [17], Pip [18], or similar.

Software and library *dependencies* should be explicit, not implicit: The package or installer should not expect dependencies to be preinstalled. Dependencies must be carefully considered to avoid “dependency hell,” whereby complex dependency graphs are difficult to maintain, especially during upgrades [19]. You should manage version numbers of dependencies to ensure future compatibility, and better still to specify compatible version ranges to avoid installing redundant library versions.

*Containerization* presents an alternative to packaged installations, as they can make dependency management much simpler by eliminating conflicting dependencies and provide a single point of deployment. The Open Container Initiative (OCI) [20] was established in 2015 by Docker to facilitate standards and interoperability. As such, it’s good practice to build your containers to the OCI specification, which can be run in Docker [21], Singularity [22], or other supported containerization software.

## Rule 3: Document your tool

Documenting scientific software is a 10-rule article in and of itself [23], so here we will make a few easy-to-follow recommendations about what documentation it would be advisable to have.

Firstly, your software project must have some high-level *project documentation*, describing what your project is intended to do and who it will be useful for. This should be short and to the point—a few short paragraphs at most. Simply put, this document should answer the 3 following questions: “What does my software do?”; “Who is the intended audience?”; “What problem does it solve for the user?”. If your software has multiple possible applications, perhaps consider documenting these high-level concerns as user stories. Diagrams, flow charts, and visualizations in documentation are highly valuable to users to manage their expectations and as an aid to knowledge transfer.

Secondly, your project must include *interface-level documentation*. Describe the purpose, data types, and units of all every method, input, and output the software exposes. This description should exist in your code repository, as a command line output (exposed via--*help*), and also include version information (exposed via--*version*) as changes to your version may result in changes to the interfaces. Ensure documentation is versioned in line with software releases.

You should describe in what *context* the tool was conceived and developed. This makes it clearer what a user might expect (and can’t expect). New features, changes, parameter defaults, and bug fixes should be documented in a *change log*. Breaking changes (particularly those that change interfaces and defaults) should be clearly and explicitly marked as breaking changes in a change log.

You should provide *code snippets* that do commonly performed tasks, including providing test input data and expected output. Ideally, these should cover all entry points and user stories. If you have optional inputs, these examples should cover minimal and maximal examples. In addition, including some minimal *test files* will aid integration into a workflow management tool.

When your tool is in a package manager (see Rule 2), you should ensure that the documentation within the package manager is up to date and appropriately versioned. It is prudent to bundle documentation (such as README.md files) along with installed software. If your software is open source, you should actively encourage your users to update documentation as well as code.

## Rule 4: Make your tool maintainable

The nature of research can at times be contrary to the nature of software development. Research is, by its nature, a moving target, and the time invested to harden and maintain software is often curtailed by the time pressures of limited grant funding. Software funded by research grants presents particular challenges for long-term *sustainability*, as grant funding typically does not allow for long-term nurturing of a software tool in the way a commercial software house would. As such, these recommendations are intended to get your project to a stage where it can “live in the wild” at the end of the project. As workflows are long-living objects, the tools within them need to be maintained for continued usability.

First and foremost, your software project must use *source control* software, such as Git [24] or Subversion [25]. It is outside of the scope of this article to explain the use of source control in detail, but it is strongly recommended that you choose a good practice guide for your particular source control system such as Git Flow [26] or GitHub Flow [27] and follow it consistently. Ideally, source control should not be hosted on private or institutional websites, as these are likely to fall into disrepair or disuse. Using a collaborative source control hosting service such as GitHub [28], GitLab [29], Bitbucket [30] or similar is highly recommended. These tools provide facilities such as access control, release management, automated building and testing, and issue tracking that will assist you in complying to the following recommendations, as well as providing a simple and well-known entry point for potential collaborators to join and maintain your project.

For maintainable software, you must use version management. While different versioning schemes exist, it is simplest to manage releases with *semantic versioning* [31]. With semantic versioning, the development path of your software can be described using just a few digits (for example, *1*.*3*.*0*), representing major version changes, minor version changes, and patches, ensuring reliable behavior for your users so long as you follow a few simple rules. Just by looking at the version number, users should know if a different version of your software will work within their existing workflow.

Your software should be downloadable as a *source code archive* or as an *installation package*. Installation packages should be kept available for all previous versions wherever possible. Where possible, bug fixes and new features should be released as new versions in a timely manner, and your community of developers should respond to user queries and issues. If funding, goodwill, or community engagement dry up, your project documentation should make clear that responses and updates are unlikely, although you should leave code open to use and allow others to freely build further on it. To this end, you should encourage formation of a *community* to respond to user queries and issues and to maintain the tool over time. This is as much about soft skills as it is about technical skills.

*Licensing* dictates how software may be used and modified. You should use an OSI-approved Open Source license [32], and ideally one that is widely known and understood, such as Apache 2 [33], BSD 2-Clause [34], or GPL 3.0 [35]. If multiple licenses are used within the same code base, you should use SPDX identifiers [36] in your comments and/or detailed LICENSE/NOTICE files.

Your source control should include any *Unit and Integration tests* using a framework conventional to the language, along with test input data, and expected output data. If your tool requires any build process, for example compilation, then you should *automate that build process*, as well as automating the running of any tests. Compiling from source code should yield equivalent output as any official installers.

## Rule 5: Follow the principle of least surprise

“*A program should follow the* ‘*Law of Least Astonishment*’. *What is this law*? *It is simply that the program should always respond to the user in the way that astonishes him least*” [37].

When your software is implemented as a workflow tool, it is explicitly with the purpose of composing it with other software tools. Tools that don’t act like users would commonly expect are likely to cause frustration and limit productivity [38]. While it could be appropriate for a specialized tool to diverge from conventions if well documented (Rule 3), users should not be required to fine-read the user manual to run the tool in its most common use cases.

Inputs to and outputs from your tools should be clearly and explicitly named, and your tool’s behavior should match your documentation. In cases where conventions (be they domain-specific or general computing conventions) exist, you must follow them as closely as possible. For example, your STDOUT stream should not include log/error output interleaved with the data output, rather logs/errors should be sent to the STDERR stream (unless configured otherwise). If your input accepts a particular file format, it should ideally support any files conforming to that format; if the tool deliberately only can support a specific subset of this format, your documentation should clearly state this.

Similarly to using standard streams in an accepted manner, you should use *exit codes* in a normal manner. If your command line software doesn’t currently use them, the simplest solution is to make sure to use exit code 0 for a successful run, and 1 for any failure. If you use exit codes other than 1 and 0, make sure to use reserved exit codes [39,40] correctly. Many programming languages provide compile-time constants, which can help with this, and can be used to ensure portability between operating systems.

Input and output files should use an appropriate community-accepted file format wherever possible, or when one does not exist, a simply parseable markup language such as JSON or XML. Even better is a file format that can easily be validated (and has an available validator). No matter how well you write your documentation, devising proprietary file formats to suit your purposes is rarely a good idea. Text output should use standard international character sets such as UTF-8 [41].

While these recommendations are not exhaustive, they should paint a picture of software that users can use without a steep learning curve. See also recommendations for writing robust command line software [5,42]. Developers are understandably often blind to the peculiarities of their own software, and this underscores the importance of having other people test your software. You will never truly know if your software is portable across different compute environments until it is let out into the wild. It will help you in the long run to make your tool open to the wider community as early as possible.

## Rule 6: Make your tool parallelizable

One of the key advantages of workflows is their ability to multiply the output of a given researcher. While automation of manual tasks increases productivity and removes human error, a workflow engine also allows parallelization of workflow runs or even workflow steps. As such, a workflow engine can potentially allow enormous horizontal scalability on even a modestly sized HTC cluster by running multiple instances [43,44].

However, when a workflow invokes software that has been designed to run as a single instance on a desktop computer, some reengineering may be required. *Fermi estimation* on order of magnitude can help with this [45]; ask yourself: “What would it look like if I ran tens, or hundreds, or thousands of instances of my software at the same time?”

A common problem is that multiple copies of the running software overwrite one another’s runtime file structure. You should ensure that multiple instances being run at the same time, whether on the same or different machines, should be independent: for example, unique temporary directories should be created per run. An instance of your software should never share memory space or internal variables with another instance (unless explicitly configured to do so, for example, with MPI).

It is worth pointing out that the task of making a tool’s *algorithm* parallelizable (for example, multithreaded) can be challenging and require significant rewrite and thread safety auditing of a tool’s code, particularly if preparing for Exascale scalability [46]. Although this can be easier in languages with strong support for concurrent programming (for example, Go, Rust, Haskell, Clojure), often sufficient scalability of a tool in a workflow can be achieved by simply catering for concurrent execution in separate processes (commonly termed “embarrassingly parallel” [47]). Tools that do use multiple threads (including by runtime libraries like java.util.stream) should ensure that they do not automatically saturate their use of machine resources, as combined with concurrent executions this could cause a counterproductive slowdown of a workflow (for example, trashing).

Machines used as nodes on HTC clusters are generally shared occupancy, and a user of your tool may well have to specify how many cores it will use when they submit a job to the job queue (see Rule 7). Likewise, if your tool accesses external APIs, there needs to be a way to keep such requests within access limitations of both server and client: The machine your tool is running on may only support a limited number of SSH connections or HTTP requests.

## Rule 7: Make your workflow tool a good citizen

Extra care should be taken when designing software likely to be run on shared infrastructure rather than on a single user’s computer—your software could potentially cause problems for other users as well as those running your tool.

Your software should be reasonably performant and should not reserve more system resources than they need. A tool that forces all cores of a processor to 100% and reserves multiple gigabytes of RAM is likely to cause issues for other users using the same infrastructure. Sometimes software is necessarily resource heavy—this should be flagged visibly to users, and the tool’s documentation should provide info on estimated resources for usage. If a particular parameter, or combination of parameters, could make your software more resource intensive, then the documentation for that parameter should make this clear. Ideally, resource usage, such as number of threads, should be parameterized (see Rule 6).

Think carefully about what you write to disk, and specifically any increases to disk usage postrun (this could mean very large output files or large numbers of small files). Your software should clear up any temporary directories it has created after it has completed successfully (perhaps with a runtime argument allowing retention of these files for debugging), and instead of internal logging, you should send any error or warning output to STDERR. Your software should not need any elevated permissions and should run correctly with the minimum of privileges.

## Rule 8: Make output reproducible

While the core tenet of empirical science is reproducibility, it has become apparent in recent years that computational methods often fall short in this regard [48]. Given the deterministic nature of computing, and the growth of resources to publish datasets and the computational tools used to process them, there is clearly room for improvement about informatics communities. One again, this is the topic of another 10 simple rules paper, which elaborates on this further [49].

The core principle here is simple: Does your software tool make it simple for researchers to include all of the data, methodology, and software tooling to allow another researcher to recreate research findings? The reality of this, though, is more challenging than it might seem. Volatile library dependencies, incomplete logging, random number generation, and many other factors can make it difficult for a researcher to replicate findings made using your tool [44].

Ideally, reproducibility should be down to the byte level. Using a hash algorithm to create checksums for file verification [44,50] is simple to implement and easily learned and allows automation of checking results. Output is often serialized as text, so be sure that your serialization is consistent across environments. You must consider whitespace normalization, sorting, local variation in decimal/thousand separators, and serialized decimal numbers should be truncated to avoid floating point errors.

Version control of your software, including managing its full dependency graph [49], is critical to maintain reproducibility. A user of your software might, for example, install your tool with a different version of an underlying library that alters output, subsequently producing unreproducible findings. If this proves to be a persistent issue, consider the portable install option in Rule 2.

Customized configuration files, especially those that are system-wide, may be inimical to reproducible output. While logging all values from the configuration file may provide all of this information, this relies on those using your software actually publishing log files, or at least storing them long term. It is best, if possible, to expose all inputs that would be stored in configuration files at runtime.

If your software relies on random seeds, these seeds must be set at run time [49]—you cannot rely on users to set them otherwise. Your software should never be allowed to persist state between runs—especially by self-modifying or referring to previous runs’ output—see guidance on atomicity of instances in Rule 6. Where persistent identifiers exist for data, they must be preserved, ideally as digital object identifiers (DOIs) [51].

Making sure your software’s output is reproducible will ultimately require testing. Multiple users must test your software on multiple machines of different configurations [48], and you should verify that the hashes of these outputs match.

## Rule 9: Carefully consider human interaction

Automation is a double-edged sword. Consider the fundamental reason for using a workflow at all: You want to minimize human handle-cranking for your computational work, yet you want that automation to be simple and human-accessible, so you don’t just want to use a script. In Rule 1, we talked about how a tool must interact well with a machine, but here we focus on how and why a tool might interact with a person.

Many tasks can, and should, always be performed computationally, such as calculation, data transfer, and so on [44]. However, tasks involving *judgment*, such as classification and interpretation, are often performed better by humans. The key cost of automation is that wrapping a tool for a workflow can end up hiding details and configurations, but also imperil visualizations and interactions with users. Scientific workflows often have some steps that don’t have a single way forward—for instance, human interpretation of the end results of workflows would be very common, but this can also happen after intermediate steps [52] where users can adjust parameters to steer the rest of the workflow [53].

If you are considering human interaction during a workflow run, you must decide if this actually adds value. There are good reasons why you would not want people involved, as this sacrifices automation and reproducibility, and can also end up producing a process where the human is the weak point. However, in many domains, workflows are not just about chaining tools together. For example, a geotagging workflow might be able to resolve a string into coordinates on a map, but to apply a sensible uncertainty radius might be best left to a human that can take the geographical surroundings into consideration.

If your workflow system does not easily support graphical interaction, a simple solution can often be to break the workflow into 2 distinct phases, where the user can make decisions at the intersections between each phase. Here, it is important to ensure that workflow outputs include both *understandable* data to inform the user decisions (for example, a HTML report) and *structured* data to reliably become inputs of subsequent workflows (for example, JSON). As a tool developer, it means that your tool should support both types of outputs concurrently (or, considering Rule 10, generating one from the other).

**Fig 1:**
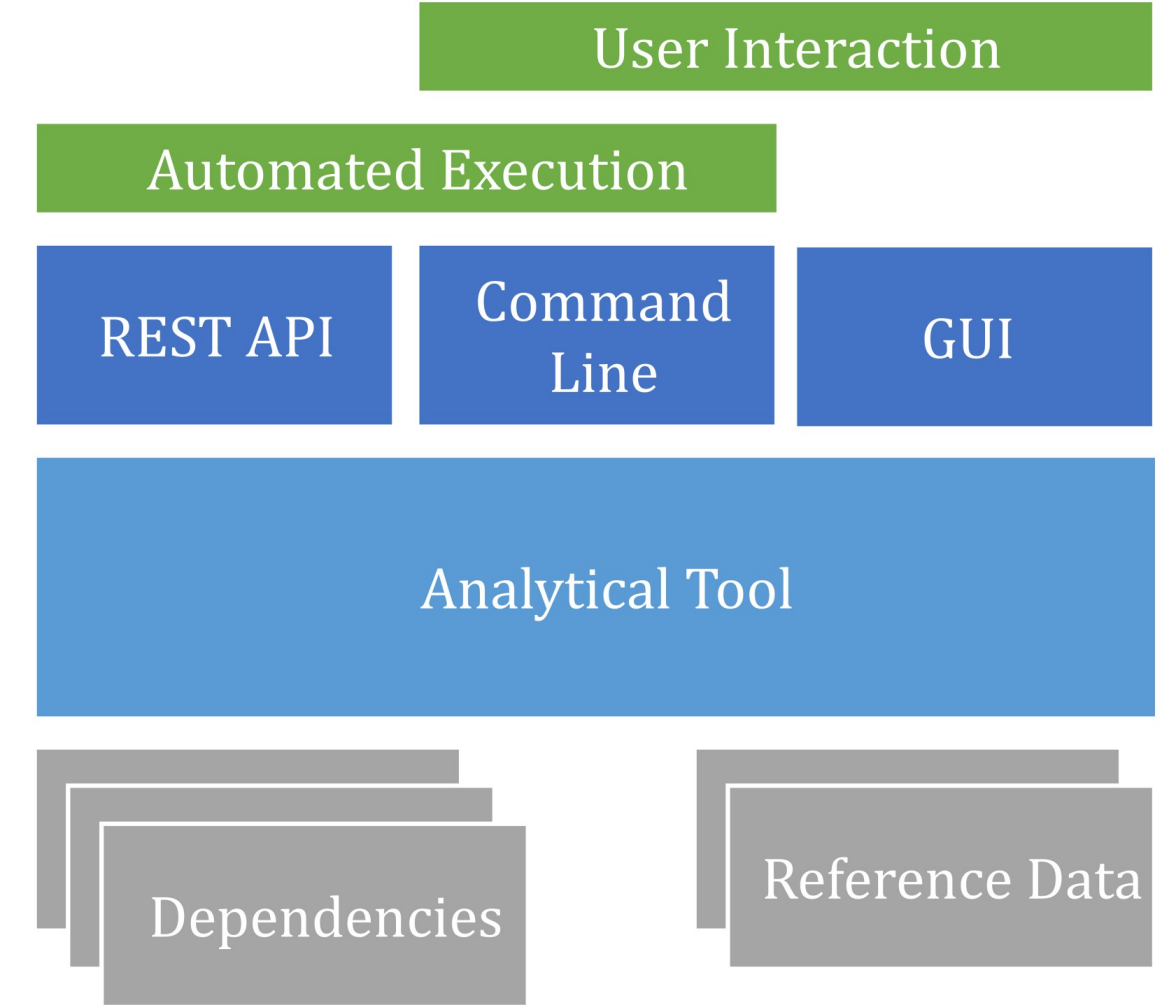
An analytical tool can be adapted for both automated execution and user interaction by refactoring the graphical and command line interfaces as separate modules that use the analytical algorithms as a common library. Such separation can also support a REST API, which can be used for both automation and interactive web interfaces.

Human interaction, however useful in some cases it might be, should not be used as an excuse to make software poorly automated—you should not create software that forces automation of its own GUI, as this is likely to be unreliable and irreproducible. This means that an application initially developed with a graphical interface may need to be *refactored* to split out a separate command line tool (Rule 1) as exemplified in Fig 1.

Any user inputs during a tool’s execution must be reported as outputs or logs, so the human interaction is made accessible as provenance [54] and potentially automatable as tool configuration or inputs.

## Rule 10: A software tool should just do one thing

The key purpose of workflows is to allow the composition of software tools. Indeed, your software is likely to have some internal composition, with multiple classes and interfaces, and it’s possible that it involves multiple steps of processing internally.

Within software design *separation of concerns* is a fundamental design principle [55], but developers may only address it within a piece of software, instead of considering the boundaries of the software tool itself. If you have gone to the effort of effectively separating concerns within your tool, it’s worth asking—would other developers find any of those internal tools useful if they were independent? If so, it might be worth turning your software into multiple tools. This is known as the *single responsibility principle* [56].

Consider the example of a tool that first reads large amounts of data from disk, converts the files to another format, before loading it into memory and performing a computationally heavy analysis. It’s quite possible that such a tool would use system resources much more efficiently if split into its component parts before implementing on a HPC, by not reserving processor or memory resources while performing I/O bound work. Indeed, this concept is enshrined in the “Unix philosophy,” which most tools used on HPC systems follow: “write programs that do one thing and do it well” and “write programs that work together” [57].

In cases where your software can perform several discrete tasks, consider that you could expose your executable with several wrappers and implement the multiple endpoints as different workflow tools. This is an ideal option if your software performs a variety of functions and allows a separation of concerns while still allowing broad functionality in a single codebase.

## Conclusions

The increasing maturity of workflow software and the rapid growth in their uptake presents a crucial opportunity to creators of scientific software. The challenges of how to make software suitable to use within a workflow engine may seem daunting; however, we argue that it can be sufficient to apply a few easily achievable measures. These 10 simple rules are intended as a first port of call for those implementing their tools within a workflow engine and seek to promote good practice across the software development life cycle. Implementing all of these rules at once isn’t necessary—software development typically works best as an iterative process, so it might be best to work through the rules one by one as guidance. Always remember: Perfect is the enemy of good.
